# Female migrants into Fukushima: A qualitative approach to their migration-support needs after the nuclear accident

**DOI:** 10.1371/journal.pone.0309013

**Published:** 2024-08-16

**Authors:** Tomoyuki Kobayashi, Tomoo Hidaka, Rie Mizuki, Akemi Kobayashi, Masaharu Maeda

**Affiliations:** 1 Department of Disaster Psychiatry, Fukushima Medical University School of Medicine, Fukushima, Japan; 2 Department of Hygiene and Preventive Medicine, Fukushima Medical University School of Medicine, Fukushima, Japan; 3 Radiation Medical Science Center for the Fukushima Health Management Survey, Fukushima Medical University, Fukushima, Japan; Japan Atomic Energy Agency / Ibaraki University, JAPAN

## Abstract

We aimed to examine the support needs of women who migrated to Fukushima Prefecture after the Fukushima Daiichi Nuclear Power Plant accident. In recent years, the presence of migrants has become an important part of the government’s reconstruction policy for affected areas. However, there is insufficient research on the status of migrants in these areas, and it is unclear what kind of support the migrants, especially females, require to encourage further migration to the area. We conducted three semi-structured interviews each with four women who had migrated to Fukushima after the accident. The narratives obtained from the interviews were summarized into categories through open coding and were finally presented as support needs in the form of a four-quadrant diagram. Four needs were identified for female migrants in areas affected by the radiation disaster: “soft adaptation needs,” “lifestyle constancy needs,” “female empowerment needs,” and “community participation needs.” Female migrants in the affected areas may be marginalized in terms of receiving migrant support. Key strategies for supporting female migrants in radiation disaster areas include providing an environment in which they can relax, interact with Fukushima and its residents, and address intersectionality.

## Introduction

During Japan’s recovery from the Fukushima Daiichi Nuclear Power Plant accident that occurred in March 2011, support for migrants who came from outside the prefecture received increasing attention [1]. These residents, who migrated to the disaster-affected areas, play a crucial role with respect to rebuilding of local communities in affected areas, residents who migrated after the disaster are important members of these communities. They have an even greater role in the affected areas of Fukushima Prefecture, where the rate of return of evacuees has not increased [2]. The Japanese government’s basic policy for reconstruction also addresses encouraging more individuals to migrate.

However, adequate support measures for these migrants have not yet been developed because their status is not sufficiently clear. Women requiring disaster support are likely to be particularly vulnerable owing to inequitable treatment associated with, for example, traditional gender roles [3]. Although these women require special consideration, the existing support for migrants [4] lacks gender-based differentiation.

### Migrants in radiation disaster recovery

The main target of disaster recovery efforts is the affected people or affected area. In most natural disasters, it is assumed that the affected people who are evacuated eventually return to their hometowns; in this case, support for affected people is consistent with support for the affected area. In radiation disasters, however, the affected people tend to be evacuated for a long time and are less likely to return to their hometowns. In 2023, the Reconstruction Agency of Japan [2] conducted a survey of the affected people in Tomioka, one of the areas affected by the 2011 Fukushima disaster. Only 10.9% of the affected people returned after the evacuation order was lifted, while 48.3% of the affected people did not intend to return in the future. The Japanese government implemented decontamination measures covering as much of the designated evacuation zone as possible in accordance with the Act on dealing with radioactive material contamination; seven years after the accident, decontamination was completed for the whole area, except in zones designated as difficult to return [5]. A major reason for people not intending to return was that, in addition to the unclear future of their hometowns and insufficient medical and welfare resources, the evacuees had already established new livelihoods in the areas to which they had relocated. Therefore, because many affected people now had routine lives outside their hometowns and because supporting these people did not lead to support for the affected areas, a gap in support occurred.

It may not be possible to reconstruct the original communities in the affected areas because of the low number of returning members. Therefore, new communities need to be created in these affected areas to reconstruct the soft infrastructure. Thus, migrants from outside areas can be envisioned as members of these new communities. In fact, in its second reconstruction and creation plan, the Japanese government identified the promotion of migration and settlement as one of its reconstruction measures, suggesting that the presence of migrants should be part of recovery efforts [1]. In July 2021, Fukushima Prefecture established a center to support migration to affected areas with the aim of promoting settlement [6]. As support for affected people has been consistent with support for affected areas in disaster recovery in the past, the spotlight on migrants from outside areas as targets of support for disaster recovery may be a phenomenon unique to radiation disasters, where the rate of return of affected people is low despite successful decontamination.

However, we have insufficient understanding of the status of migrants from outside areas with regard to recovery from the 2011 Fukushima disaster. In the context of recovery, these migrants are often perceived as not requiring support. One reason for this lack of understanding is the importance accorded to dealing with radiation exposure–related problems as a countermeasure to the radiation disaster. In the recovery efforts, the spotlight was on the affected people and evacuees who were at risk of radiation exposure [7]. From this perspective, the focus is less likely to be on migrants, as they may not have been exposed to radiation at the time of the accident (except for decontamination workers involved in nuclear power plant operations). Alternatively, our lack of understanding may be because studies examining the circumstances of the disaster have been methodologically unable to distinguish between migrants and affected people. In fact, epidemiological and social science studies on the effects of the Fukushima nuclear accident have often excluded post-accident migrants from their analyses or have included them only as part of a control group of unaffected people [8–10].

At present, support for migrants often focuses on helping them start a new life or obtain childcare or employment [4], with an emphasis on encouraging people outside Fukushima to migrate. However, such help may not be sufficient for people who have moved to the affected areas to settle down. These areas are engaged in addressing radiation issues that are unfamiliar to migrants, and there is serious stigma regarding radiation exposure [11]. Migrants are concerned that this stigma will be directed toward them. Moreover, the migration process can involve the risks of various psychological and social problems, such as changes in the living environment, breakdown of friendships, and loss of economic foundations [12]. Thus, both research and support efforts must consider the fact that a lack of risk of direct exposure to radiation at the time of the accident does not guarantee that there will be no anxiety about living in the affected area.

### Migrant support and gender issues

Marginalized populations often receive inadequate support despite their vulnerability. Due to the different accessibilities of each target population, superficially equal support does not result in equitable benefits. Therefore, disaster support, including support for migrants, must identify those who are marginalized in society and approach them discriminatorily. Female migrants are more likely to experience disadvantages than male migrants [13].

One disadvantage faced by female migrants is the primacy of male careers in migration decisions. Brandén [14] conducted a survey of married or cohabiting men and women and found that women were more willing than men to migrate for their partners’ careers. Migration involves the loss of social ties and careers gained in one’s previous life circumstances. If male careers are prioritized over female careers during migration, the risk of career loss will be greater for women.

Women also experience a higher risk of discrimination based on traditional gender roles after migration. For example, women are required to find new employment after their careers are cut off by migration; however, they are more likely to face discrimination based on gender roles in their career development and in seeking jobs with childcare and family management in mind [15].

In addition, women are more anxious than men about nuclear accidents [16]. The effects of radiation are often discussed in terms of pregnancy outcomes and the health of future children, leading to the stigmatization of affected women and their peers [17, 18]. Therefore, young women from Fukushima often attempted to hide the fact that they once lived in Fukushima, especially in the early years after the accident [19]. A survey of mothers with infants in Fukushima found that the likelihood of depression was higher among women living in affected areas [20]. Although these surveys did not focus on female migrants, they suggested that living in affected areas is likely to lead to stress in childcare, which presents a variety of challenges.

Women’s vulnerability to migration and the effects of radiation disasters are, therefore, likely to create livelihood difficulties in migration to affected areas, suggesting that gender disparities are likely to emerge in the availability of childcare- and employment-related support in migration support services. Careful consideration of women’s support needs is necessary when formulating support measures.

In previous disaster studies, there has been limited focus on female migrants in affected areas, although their support needs may differ qualitatively from those of affected people. Therefore, in this exploratory study of the support needs of female migrants to the affected areas, we adopted a qualitative approach because of the novelty and specificity of the subject.

### Purpose of the study

To address the aforementioned lack of understanding of and support for post-disaster female migrants, we aimed to examine the specific support needs reported by female migrants in areas affected by the Fukushima radiation disaster.

## Materials and methods

### Research design overview

We conducted longitudinal semi-structured interviews with four women who migrated to Fukushima Prefecture after the Fukushima Daiichi Nuclear Power Plant accident. Participants were recruited through a support group for female migrants and were selected via purposive sampling. Three interviews, all of which were recorded, were conducted with each participant. We created verbatim transcripts based on the recordings and analyzed them using an open coding approach to summarize the narratives in a descriptive manner.

### Researcher description

The interviews were conducted by two people: a social psychologist and a clinical psychologist. Both were university faculty members with extensive experience in conducting interviews. One was male and one was female, and both had moved to Fukushima from outside the prefecture. Two participants were assigned to each interviewer.

Two other university faculty members—a social psychologist and a public health nurse—joined the two interviewers in performing the analysis. One was male and one was female; one lived outside Fukushima and one had moved to Fukushima from outside the prefecture.

### Participants

Four women who had migrated to Fukushima Prefecture after the 2011 Fukushima Disaster participated in the interviews. The required sample size for this study was based on the study by Sato and Fukuyama [21], which discussed the number of interviewees based on their heuristics, suggesting that four interviewees can suitably portray the diversity of experiences of the target group.

We asked organizations that supported female migrants in Fukushima Prefecture to cooperate with us in recruiting female migrants after the disaster. The head of an organization posted a recruitment poster created on a Facebook group page that the organization usually uses to communicate with its members. The organization was founded in 2020, and its members did not necessarily join immediately after moving to Fukushima. None of the participants had met the interviewers or analysts before participating in the study.

### Data collection procedures

Between October 2022 and March 2023, participants visited individually scheduled interview rooms to participate in the survey. The interview room included a computer was located in the interview room, using which the participants accessed Zoom, an online communication tool.

All the participants provided informed consent to participate in the interviews. To ensure that they felt comfortable participating in the interviews, they were allowed to remain alone in the room during the interview. However, one participant had given birth during the study period and was accompanied by her infant. She was free to care for the child during the interviews, as needed. In addition, a staff member was stationed outside the interview room to address equipment problems.

Table 1 presents the stages of the study. The interviews were conducted by a single interviewer. Each longitudinal semi-structured interview lasted nearly three hours, and each interviewee participated in three interviews. The initial questions covered the life history of the respondents from before they moved to Fukushima to the present. Although the focus was on the process of migration and the difficulties the respondents faced in their daily lives in Fukushima, an interim analysis was conducted after each interview to organize and update the interview content. To ensure that the results compiled by the researchers in their analysis did not diverge from the participants’ narratives, the findings from the interim analysis were fed back to the participants in the next interview session, and their opinions were sought. The interviewers had the flexibility of adding questions and asking for more details based on their responses to the feedback. The feedback from the analysis had two aims: to enhance the validity of the results. The first was to modify the analysis results if the narratives were misinterpreted due to analysts’ biased perceptions and opinions. The second was to confirm that the narratives were not spurious by asking participants about their own and others’ narratives. The interviewers also asked if participants had experience with the needs of issues found in general migration (e.g., economic changes and interaction with the community) during the second interview. The interview guide is presented in detail in S1 File.

**Table 1:** Stages of the study: Interviews and analyses.

Stages	Task	Contents
1	Interview 1	Semi-structured interview about how they migrated to Fukushima
2	Analysis 1	Open coding for each participant
3	Interview 2	Feedback on Analysis 1 results
		Additional questions about the process of migration to Fukushima
		Questions about needs found in migration in general
4	Analysis 2	Open coding for each participant
		Summary of all participants’ codes. Tentative modeling
5	Interview 3	Feedback on Analysis 2 results
		Additional questions about the process of moving to Fukushima
6	Analysis 3	Open coding, one participant at a time
		Summary of codes for all participants. Final modeling
7	Feedback	Feedback on Analysis 3 results

Participants received a payment of approximately $100 (13,700 yen) per interview. All interviews were recorded with the permission of the participants.

### Data analysis

The narratives recorded during the interviews were transcribed verbatim. The analysis was conducted using the open coding method [22], in which the narratives were summarized in a descriptive manner. This analysis method involved the following steps: First, the narratives in the verbatim transcripts were intercepted as a single unit of speech or dialogue. Subsequently, all intercepts were provided with a short summary indicating their content. Next, the intercepts with summaries were grouped based on their similarity in content, and labels were attached to the grouped intercepts to indicate their contents. The grouping and labeling processes were then repeated to gradually abstract the contents of the labels.

Coding was first performed individually for each participant, and the entire model was created in the second and third analyses. The second and third analyses were reconciled with previous coding results. These coding tasks were performed using pens and sticky notes.

### Methodological integrity

We examined the status and migration needs of post-accident migrants to areas affected by the nuclear power plant accident. Previous disaster research has focused mainly on the affected people and evacuees living in Fukushima at the time of the disaster; therefore, post-disaster migrants have been overlooked in these studies. In Japan, women are often left to take care of housework and childcare, and they were likely to have faced problems with employment and the development of their living environments when they migrated with their families. These women may be socially marginalized in the context of immigration.

This study fulfilled the appropriate methodological requirements for data collection and analysis. We were able to select a sample of individuals who met the attributes of “female migrants” and who were potential providers of rich narratives; moreover, we were able to inductively analyze and categorize support needs by using open coding. Our findings provide important and novel lessons for disaster research and contribute to a better understanding of post-accident migrants and the recovery of affected areas following the nuclear accident.

However, as participants were recruited through support groups for female migrants, they may have already received some level of support (e.g., alleviation of isolation and accessibility to information). Moreover, all participants had migrated after the disaster with the knowledge that Fukushima was affected by the nuclear accident. In other words, these women did not abandon their migration because of concerns about radiation risks. In this regard, they may be independent enough to ignore the opposition of their friends and parents in their hometowns or they may not be independent enough to accommodate their husbands’ requests.

To avoid bias in the interpretation of the data and to avoid reflecting on the experiences and ideologies of specific individuals, a specialist in qualitative research participated as a mentor and analyst, and our four interviewers and analysts had a variety of professional backgrounds. Although not all of these were always able to participate in the analysis sessions, those who were unable to participate were kept informed separately and appropriately of the progress of the analysis. After each analysis, the results were shared with the participants themselves to verify that the degree of abstraction and consistency of the procedures were maintained and to ensure that the linguistic expression of the label names accurately represented the content. Since all the researchers in this study moved to Fukushima Prefecture after the disaster, there was a concern that their own needs as migrants might be reflected in the results. We made careful assessments to ensure that the results of the analysis were not biased by the researchers while providing feedback to the participants themselves.

### Ethics

This study was conducted with the approval of the Ethics Committee of the Fukushima Medical University (Approval No.: General 2022–120). Prior to the interview, the interviewer obtained informed consent both verbally and in written form. After explaining the details of the research and the methods involved, the participants provided their written consent by signing a consent form, which was provided to each participant. The informed consent procedures were captured through audio recordings to verify the process.

## Results

### Participant characteristics and coding

Table 2 shows the demographics of the participants, the schedule, and the duration of each interview.

**Table 2:** Participant characteristics and schedule of interviews.

	Gender	Age	Residential area	Reason	Migration year	Interview 1		Interview 2		Interview 3	
						Date	Duration	Date	Duration	Date	Duration
P1	Female	30s	A pref. → B city	Marriage	2016	October 16, 2022	2 h 54 m	December 21, 2022	2 h 25 m	2023/2/17	2 h 44 m
			→ A pref. → C city								
P2	Female	30s	D pref. → E city	Marriage	2013	October 20, 2022	1 h 50 m	December 20, 2022	1 h 52 m	2023/2/28	2 h 48 m
P3	Female	40s	F pref. → G city → C city	Marriage	2015	October 24, 2022	2 h 51 m	January 11, 2023	2 h 21 m	2023/3/14	2 h 48 m
P4	Female	40s	H pref. → C city	Marriage	2011	October 31, 2022	2 h 50 m	December 27, 2022	2 h 43 m	2023/3/2	2 h 36 m

**Note:** Residential areas are anonymized and are indicated by “city” if inside of Fukushima Prefecture and by “prefecture” if outside Fukushima Prefecture.

P, participant number

Stepwise coding resulted in three or four coding rounds per participant (Table 3). Each participant yielded five to nine categories, and individual categories were combined to create a final total of eight categories describing the status of female migrants.

**Table 3:** Number of intercepts and categories for each phase in open coding.

	Interview	Intercepts	Coding round per interview				No. of coding categories per participant	Total no. of coding categories
			1	2	3	4	5	6
P1	#1	192	39	13	6	-	7	8
	#2	112	69	26	10	-		
	#3	116	66	23	6	-		
P2	#1	101	32	12	5	-	5	
	#2	107	75	26	9	-		
	#3	96	57	22	11	4		
P3	#1	118	56	21	9	6	8	
	#2	106	44	11	5	4		
	#3	77	41	15	5	-		
P4	#1	70	39	14	5	-	9	
	#2	154	68	23	9	7		
	#3	104	49	19	7	-		

P, participant number

### Support-needs model for female migrants

**Fig 1:**
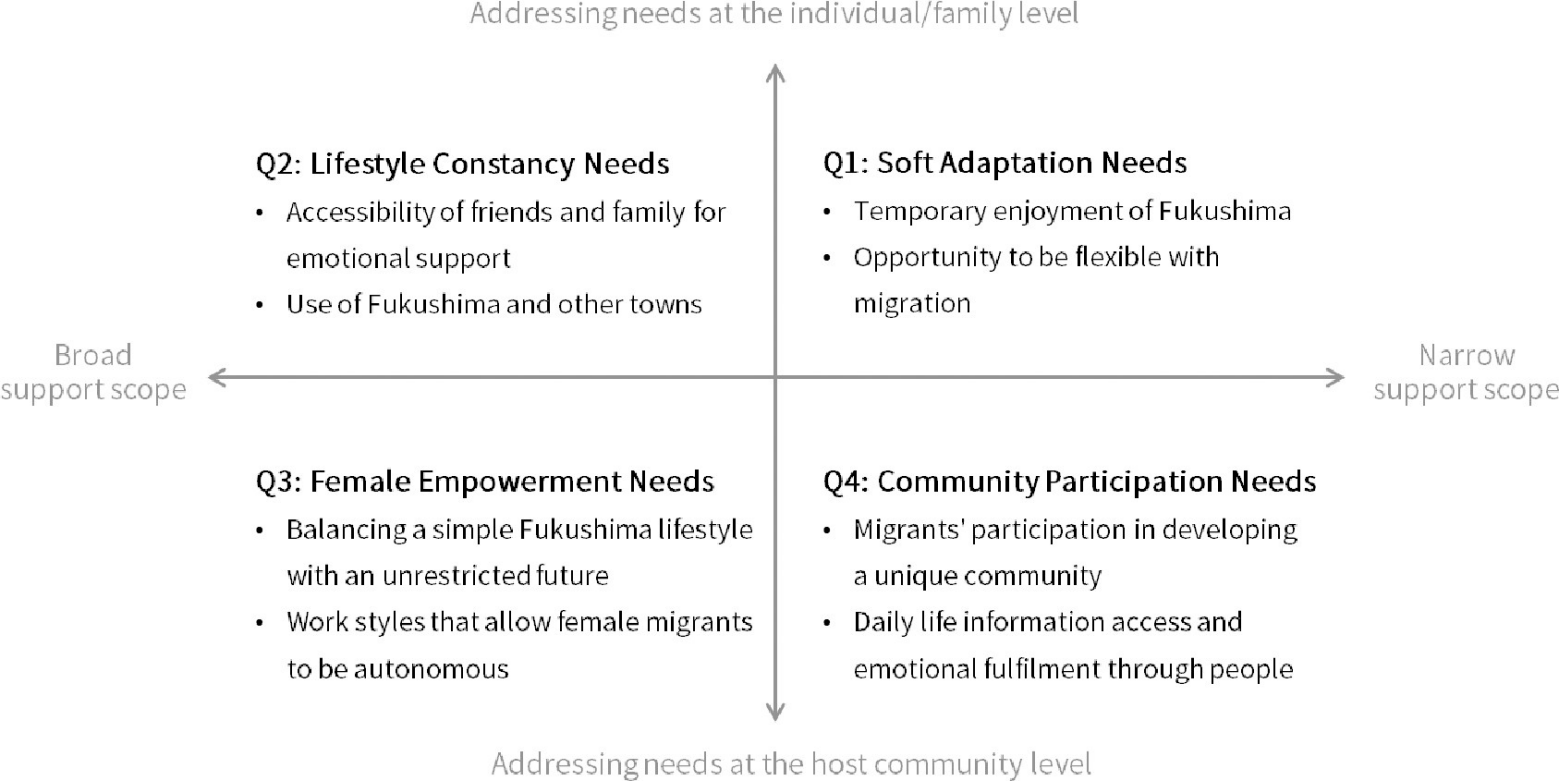
Support-needs model for female migrants in nuclear-disaster-affected areas.

We created a support-needs model for female migrants using a four-quadrant diagram that summarized the eight status categories (Fig 1). The diagram has two dimensions: a vertical axis representing the unit of support needs at the individual/family and community levels and a horizontal axis representing the scope of support needs, with a narrow focus on Fukushima and a broad focus that included Fukushima.

The first quadrant, labeled “Soft Adaptation Needs,” encompassed categories such as “temporary enjoyment of Fukushima” and “opportunity to be flexible with migration.” These needs focused on Fukushima and were addressed at the individual or family level in the narratives. The narratives featured female migrants who appreciated life in their new locations but did not identify themselves as permanent residents of Fukushima, desiring soft connections that allowed for the possibility of leaving at any time. The following narratives were included:

Fukushima is Fukushima, and my hometown is my hometown; I have noticed that the way I enjoy my life here is different from the way I did. I’ve begun to think about how I can enjoy my life here in a way that is different from how I enjoyed it in my hometown. (P1)

I try not to talk for long. I want to listen to other people’s stories, because Fukushima’s stories seem more interesting… I don’t feel like it’s hard to talk to them at all. I prefer to listen to what others say I wonder if this is what Fukushima is like, the dialect: What is this kind of thing called? I think it would be interesting to hear them talk like this. (P4)

The second quadrant, labeled “Lifestyle Constancy Needs,” encompassed categories such as “accessibility of friends and family for emotional support” and “use of Fukushima and other towns.” These needs focused both on Fukushima and outside the prefecture, and were addressed at the individual or family level in the narratives. The female migrants believed that migration would not essentially change their lives, although their environments will change superficially. The narratives featured a desire to develop their living environment without having to focus on completing their lives in Fukushima. The following narratives were included.

When I was in city A [urban city name anonymized by authors], if I said there was something going on somewhere on the weekend, I’d be like, “Let’s go together” with friends. At night, we’d go out to drink. Even if it were not a drinking party, we would go out to eat. Well, that is a fairly standard style. Even after we become working adults, it’s quite normal to say, “Let’s meet after a long time,” and grab a meal together. Even if I had children, I’d still meet up with friends, and let the children play together sometimes—that sort of thing. But, in reality, that never happened in Fukushima. (P3)

Even if I lived in my hometown, I don’t think anything would really change. Just a change in residence, a change in the house I live, but life is the same. Am I making sense? It would be strange because I said that my life has changed, but it hasn’t … It has changed, but it also hasn’t changed. Do you get it? My conditions haven’t changed. My friends are not close to me, you know. That changed. In that sense, I don’t have my parents nearby, and I don’t have siblings nearby. The environment changes, but I go shopping, and that’s a constant in my life, right? So, what is it … maybe it’s the change of environment. The environment changes, but life doesn’t change … Migration means that things change. Environment? What is it? The view changes? Do you understand what I’m saying? (P2)

The third quadrant, labeled “Female Empowerment Needs,” encompassed categories such as “balancing a simple Fukushima lifestyle with an unrestricted future” and “work styles that allow female migrants to be autonomous.” These needs focused beyond Fukushima and outside the prefecture and emerged from the lack of adequate environmental conditions in the host community that could not be solved by the migrants themselves. Female migrants face career disruptions due to migration and discrimination due to childcare needs and marriage. Moreover, they did not have bright expectations for their own and their children’s futures. The following narratives were included.

It was difficult to find work after marrying and moving here. People would think I’d move again eventually, so it was harder to get hired… As a result, I was unable to find a job for a while. And I get it, it makes sense. If I knew someone might up and leave at any moment, I wouldn’t hire them either… But still, I can’t just sit at home all day; it’s boring, and I want to work. It feels like a waste, doesn’t it? (P2)

So I switched my work to freelance. I was restricted by the need to work on weekdays … [Working] fixed hours was very burdensome for a parent with small children, so I wanted to change to something else for a while. It was then possible for me to adjust my work hours, and I was finally able to attend meetings [networking groups with other mothers]. (P3)

The fourth quadrant, labeled “Community Participation Needs,” encompassed categories such as “migrants’ participation in developing a unique community” and “daily life information access and emotional fulfilment through people.” These needs were focused on Fukushima and, like those in the third quadrant, emerged from a lack of adequate environmental conditions in the host community. Female migrants had low expectations for better development of the towns to which they were migrating and communicated their demands regarding what they wanted the town to be like. However, they did not expect their opinions to be reflected in the development of the town. The narratives featured a desire to be accepted by the host community and the residents as migrants. The following narratives were included.

Anyway, the shopping locations are too poor. I’m so stressed that, even when I make it to the station area, there’s nothing I want to buy. No places I want to visit. If there was somewhere I wanted to go to, I would try my best to practice driving, or even if it took a little longer, I would go somewhere, such as the station or the store. This sense of excitement has disappeared. That’s the hardest thing for me mentally … Like, if I want to go out for coffee, there’s only Starbucks. I couldn’t choose anything else. However, sometimes, I feel like trying Tully’s or some other place, you know. The lack of choices makes it so boring. The supermarket is here, the pharmacy is here, and the coffee place is here. It’s boring … My friends who migrated to Fukushima from their hometowns also said that there were no clothing stores where they wanted to shop. Thus, they ended up buying only daily necessities and food in Fukushima; they felt their shopping was more fulfilling if they went outside the prefecture, to either Nasu or Sendai. (P1)

When I submitted our marriage registration at the city office, it was just taken and they said, “Received, congratulations!” However, it would have been nice if they could have told me about activities in the town at that time … If they had put something welcoming in an envelope and handed it to me, it might have given me a little more information. Perhaps I could have gotten to know about the tea parties organized by the people who moved in. (P4)

### Narratives related to the radiation disaster

Narratives about radiation anxiety were included in the first quadrant, “Soft Adaptation Needs,” the second quadrant, “Lifestyle Constancy Needs,” and the third quadrant, “Female Empowerment Needs.” In the first and second quadrants, the female migrants mentioned that their lack of experience with the radiation disaster made it difficult for them to talk about the accident and radiation. In the third quadrant, they expressed the need to value their children’s education because Fukushima was a disaster-affected area. The following issues were mentioned:

I thought I shouldn’t talk about radiation with the people from Fukushima … after the disaster happened, and, you know, it happened, we can do nothing about it. Like, if I said, “I’m afraid of hanging out my laundry,” it would remind them about the disaster. And there are people living in areas with high radiation doses. So I wondered if I shouldn’t talk about it too much. It’s, well, it’s because I’m not from Fukushima. I wasn’t born and raised here. I wasn’t here on the day of the disaster … So, I guess that’s why I think this way… Also, I wondered if my friends or parents would be worrying about me if I told them. (P4, first quadrant)

They are going through a terrible time. Such feelings toward the people from Fukushima … [Because I wasn’t here] I wasn’t able to pay more interest in or be closer to them. I think the good thing [about living in Fukushima] is that being here keeps me thinking about it [the Fukushima disaster]. (P3, third quadrant)

## Discussion

This study employed a qualitative approach, using semi-structured interviews to examine the support needs of women who had migrated to areas affected by the Fukushima radiation disaster. Eight categories were obtained describing the status of female migrants, and a support-needs model consisting of four needs and two axes was constructed to represent the support needs. Three of these four needs covered narratives related to the radiation disaster.

### Four needs of female migrants in areas affected by the radiation disaster

Previous efforts to deal with radiation disasters have not adequately considered migration to affected areas. Owing to the unique nature of radiation disasters among other disasters, the presence of migrants in the affected areas is an important component of support for these areas. The unique interactive and cumulative impacts of radiation disasters on migrants, especially women, must be carefully considered when considering support for female migrants in areas affected by radiation disasters.

The first quadrant of the support-needs model, “Soft Adaptation Needs,” indicated that migrants did not identify themselves with the host community but required a soft connection that allowed them to leave at any time. Disaster studies have reported that identification with the community contributes to stress reduction [23] and resilience [24]. Therefore, encouraging a connection to the community is important for helping affected people [25]. However, female migrants did not prefer a strong identification with the community, but rather preferred to be in a position where they were only visitors to the community—residents but foreign guests. This position also allowed them to enjoy life in the area of migration with ease. Berry’s model of acculturation, which discusses the acculturation of migrants, points out that it is better for migrants’ mental health to be able to successfully integrate and retain their original and host cultures rather than to retain only their original culture or to assimilate into the host culture [26]. Establishing a loose connection with the host culture may be important in the process of integrating different cultures. Nevertheless, if the migration site is an area affected by a radiation disaster, the migrant faces radiation risks. The radiation risk may have influenced the choice of acculturation strategy. First, migrants felt that they could not talk about radiation with affected people because they themselves had not experienced the disaster; thus, they were hesitant to talk about radiation with affected people out of consideration for the feelings of the affected people. Moreover, we should consider that there might be discrimination among females regarding relocating to radiation-affected areas in Fukushima. People living outside the prefecture may not positively agree with women migrating to areas with radiation risks. Criticism may come more from women than from men, as participants said that they did not want to divulge their radiation concerns to their friends or parents. Even if the migrants hoped to be entirely accepted by host communities and to be regarded as community members, they may express their feelings in a more passive way due to differences in their experiences with the residents of the community and worries about discrimination by other women in their hometown.

The second quadrant, “Lifestyle Constancy Needs,” indicated that female migrants believed that migration was not essentially livelihood changing, despite the environmental changes. Previous studies on migration have theorized that there are two types of factors motivating people to migrate from one place to another: pull factors, which are attractive factors of the destination that make the migrant want to go there; and push factors, which are negative factors that make migrants want to leave their original place [27]. For example, labor migration to obtain a better economic environment is migration based on pull factors, whereas refugee migration to escape conflict is migration based on push factors. Although this theory suggests that migrants are motivated by a desire for a change in their living environment, our study suggests that the participants believed that the nature of their lives was not changed by migration. Conversely, urban-level changes resulting from migration to Fukushima and anxiety about the areas affected by the radiation disaster were risk factors that could change the lives of female migrants. These factors may be unavoidable in areas affected by nuclear disasters, as the original community may not be able to provide adequate support in the event of its collapse. The narratives uncovered here suggest that the migrants did not restrict their pool of available resources to the Fukushima Prefecture, but instead escaped essential livelihood changes by freely accessing resources outside Fukushima. Thus, although female migrants do not seek essential changes in their lives, they maintain constancy in the process of migration by accessing resources outside the migration site.

In the third quadrant, “Female Empowerment Needs,” the women faced career disruptions due to migration and discrimination due to moving to the area to raise children or get married. They did not have optimistic expectations for their own or their children’s futures. As noted in previous studies [14], the female migrants in our study accompanied their husbands upon marriage, and all experienced career disruptions. They faced difficulties during job hunting in terms of gender-based discrimination, childcare needs, and disparities in the work environment between their places of origin and Fukushima. First, previous studies have pointed out that female migrants are less likely to have economic stability after migration than male migrants [28]. Similarly, in this study, female migrants were more likely to quit their jobs because of marriage; further, they experienced difficulties during job hunting because of the discriminatory notions held by potential employers, such as thinking that women will be busy with their households. This is not unique to Fukushima and has been highlighted in previous studies [15]. The participants also discussed the difficulties of balancing work and childcare. For example, it is difficult to attend child-rearing seminars and meetings while working, because they are often held during typical working hours when childcare is required. This makes it difficult for women to continue working if they have to prioritize childcare. Furthermore, the average wage in Fukushima is lower than in urban areas, and there are few employment options. Although this does not always cause poverty in Fukushima, because the cost of living is lower than that in urban areas, wages can be perceived as a criterion for evaluation as a worker. One of the female migrants that we interviewed experienced self-esteem wounds due to low wages.

Finally, the fourth quadrant, “Community Participation Needs,” indicated that female migrants wanted to be accepted by the town and by local residents as migrants. Migrants are physically separated from social networks in their original communities and are more likely to be isolated in their new communities [12]. To reduce this isolation, it is important for migrants to participate in the community to which they have relocated. It should be emphasized that this is consistent with the first quadrant, “Soft Adaptation Needs.” Migrants do not want to identify with a single host community; rather, they want to be involved in the host community while maintaining their original identity. The possibility of realizing a migrant’s needs depends on the destination community’s attitude toward accepting the migrant [26]. However, the participants anticipated that the host community would not be interested in having migrants join their community. Host community members in affected areas may have complicated feelings about accepting migrants. They may have been relatively accustomed to accepting outsiders because experts and volunteers from outside entered the affected areas after the accident. However, they may wish for a recovery that values the original culture of the community and often aims for restoration to the pre-disaster state. On the other hand, the migrants are not familiar with the original culture of the host community and some bring new ideas to it. Accepting participation in community development by migrants who do not know the community at the time of or prior to an accident entails the risk of changing the original culture. Therefore, the participation of migrants in the community in the affected area requires a balance between the restoration of the original culture and the risk of cultural transformation in the community.

### Practical implications for supporting migrants in the affected areas

This study identified four support needs of female migrants in areas affected by the radiation disaster, namely “soft adaptation needs,” “lifestyle constancy needs,” “female empowerment needs,” and “community participation needs.” Each of these needs requires action at the individual or community levels, involving the development of resources within the affected areas and the utilization of resources from outside the affected areas. Based on these four needs, two key strategies for support have been recommended: providing or facilitating soft connections for female migrants with the areas to which they have migrated and addressing the intersectionality of female migrants, particularly in relation to traditional gender roles.

The presence of migrants in the reconstruction of affected areas is considered important, especially in cases where the return rate of evacuees is low, as in the case of radiation disasters. Previous studies have excluded migrants for research interests and methodological reasons [8–10]. To the best of our knowledge, the actual situation and support needs of migrants have not been adequately explored. However, our study showed that even migrants have concerns about living in the affected areas. In particular, female migrants within the affected areas are highly vulnerable, even though they have not been affected by the disaster, as they have to deal with changes in living environments, isolation, discrimination due to migration, and concerns specific to the affected areas (e.g., radiation risk). When planning the reconstruction of areas affected by serious disasters, including wars and conflicts, we need to incorporate specific support for female migrants.

For reconstruction after the Fukushima Daiichi nuclear power plant accident, we propose support that extends the existing support for migrants based on the aforementioned key strategies. The existing migrant support in Fukushima Prefecture involves providing financial help for migration to Fukushima, such as subsidies for housing acquisition [29], subsidies for living expenses for migrants [30], human resource matching to specific occupations [31], and help in returning scholarship funds [32]. These measures can be expected to work effectively in easing migrants’ hesitancy in making decisions to migrate to Fukushima. In line with this, the implications of our study findings, derived from the first key strategy—providing or facilitating soft connections for female migrants with the areas to which they have migrated—are that migrants should be given more opportunities to learn about Fukushima while maintaining their connections outside the prefecture. For example, if support and events are provided with the aim of promoting cultural activities to help people learn more about Fukushima, migrants will enjoy learning about Fukushima’s culture [33]. A teleworking support program has been implemented in the Fukushima Prefecture [34], but it targets out-of-prefecture residents. This could target those who have just migrated to the prefecture to help them adjust to their new lives. When issuing various types of migrant support, attention should also be paid to accessibility issues to ensure that support and related information reach the migrants.

Regarding the second key strategy—addressing the intersectionality of female migrants—any support should consider discrimination against women and the lack of social resources for migrants with regard to employment. Intersectionality, as discussed in the previous section, refers to the interactional and cumulative effects of the multiple backgrounds of the female migrants—those who moved to accident-affected areas, mothers, and women. For example, assistance to help female migrants with paperwork and financial support to enroll their children in daycare centers would be helpful in promoting employment among female migrants. Moreover, holding social gatherings among residents—outside typical working hours—would encourage female migrants to participate in the community [35]. Further, measures that actively encourage communication between migrants and local residents are necessary. Therefore, it will be important to conduct information campaigns to demonstrate the benefits of immigrant participation in the recovery process. Several media outlets have already featured articles on how migrants contribute to the town [36]. This would also help migrants who have to deal with radiation anxiety in isolation owing to the barriers they face vis-à-vis local residents.

### Limitations

This study examined the support needs of female migrants in areas affected by the Fukushima radiation disaster. However, our study has several limitations. First, the support needs of female migrants obtained in this study require a careful assessment of their importance and representativeness. The research and analysis methods in this study focused primarily on examining the needs of the participants and their backgrounds and did not measure the social priority or severity of the issues. Moreover, although we recruited individuals who fulfilled the criteria of being female migrants, the results were biased toward those who had moved to the affected area because of marriage. Therefore, we must be cautious about the generalizability of the findings to female migrants who moved for different reasons but have similar needs. In other words, for the findings of this study to be reflected in support policies for female migrants, future research is necessary to verify their generalizability and seriousness. In addition, because we focused on female migrants, it was unclear whether the labels obtained as support needs were specific to female migrants. For example, soft cultural adaptation may be helpful not only for female but also for male migrants.

Moreover, the researchers who conducted the interviews and analyses were themselves migrants who had moved to Fukushima Prefecture after the accident. Since migrants often have potentially conflicting relationships with local residents in Fukushima Prefecture, the fact that the interviewees were also migrants made it possible for them to tell their stories in a comfortable manner. However, the interview content and analysis were possibly influenced due to leading questions during interviews and biased interpretations based on the researcher’s own needs. Although this study attempted to avoid biased interpretations by the researcher by feeding back the results of the analysis to the participants as the study progressed, it is still possible that the results of this study may be biased from the migrants’ point of view. Therefore, the results of this study, especially in terms of facts, should be interpreted as perceptions of migrants. In future research, it may be necessary to investigate the views of those who consider Fukushima Prefecture to be their hometown in relation to the narratives obtained in this study.

Finally, it is difficult to determine whether the support needs identified were based on the characteristics of the disaster or the culture of Japan or Fukushima. For example, changes in living environments, such as those at the urban level, associated with migration to the disaster area may be urban-level issues in Fukushima that are unrelated to the disaster, such as a lack of commercial facilities and low wages. This difficulty in discernment can also be considered an issue of discrimination faced by female migrants. Such needs may not be necessary in areas where beliefs about gender equality are widely shared, even if a disaster occurs. However, in areas affected by nuclear disasters, the perceived lack of community reconstruction may result in delayed and inadequate policy and infrastructure development or slow resolution of all types of problems, not just migrant-related problems due to the disaster-affected nature of the area. Future research should examine this topic with regard to other disasters and disaster areas to determine the factors contributing to support needs.

## Conclusions

This study examined the support needs of female migrants in areas affected by the Fukushima radiation disaster. We identified four needs: “soft adaptation needs,” “lifestyle constancy needs,” “female empowerment needs,” and “community participation needs.” These results were confirmed in the context of the Fukushima Daiichi nuclear power plant accident, but they could also be considered important findings in other disaster contexts. In the context of disaster recovery, the main focus has been on providing care to affected people, which makes it difficult for unaffected people, including migrants, to receive care. However, as residents of the affected area, migrants still face the same disaster risks as the affected people. In the formulation of reconstruction measures, special consideration must be given to those who are more likely to be marginalized and vulnerable.

## Supporting information

S1 File Interview guide for immigrants to Fukushima. This guide was used for the interviews in this study.
(DOCX)
