# Supplementary material for: Female migrants into Fukushima: A qualitative approach to their migration-support needs after the nuclear accident
Source: PLoS One. 2024 Aug 16;19(8):e0309013. doi: 10.1371/journal.pone.0309013 (PMC11329142; doi:10.1371/journal.pone.0309013)
Supplement: S1 File — This guide was used for the interviews in this study. (DOCX) [file pone.0309013.s001.docx]

**S1 File**

**Interview Guide for Immigrants to Fukushima**

1. This interview guide should be used after obtaining informed consent, as described in another form.

2. Each interviewer needs to consider any complaints expressed about the interview by participants. If they complain of anxious feelings or other psychological issues during the interviews, the interviewer should address these issues immediately and adequately.

3. Interviewers are allowed to choose their interview mode (in-person or video) upon agreement with the participants.

4. Interviewers may modify the question items set out below to suit the situation or context.

<Participant Information>

| Name |
| --- |
| Gender |
| Current Adress |
| Where did they immigrate from? |

# Step 1 Interview

## Ask for basic information (age, hometown, family)

## Core question: “Could you tell me roughly what you did before you moved to Fukushima and up to the present?”

## Sub-questions: The following are suggested questions. The interviewer does not have to ask them and may prioritize what the participant wants to talk about.

- What do you value most?
- Can you access food and medical care in your relocation area without any inconvenience?
- How are your relationships in your relocation area?
- What would make you feel as though you have “immigrated”?
- What did you do before you migrated?
- What was it at home that made you decide to migrate? (i.e., What were the push factors?)
- What was it about Fukushima that made you decide to move there? (i.e. What were the pull factors?)
- What were your first impressions of Fukushima when you arrived?
- What did you expect from life in Fukushima at the time you left home?
- What aspects of life in Fukushima did you worry about?
- What information did you refer to when you were thinking of moving to Fukushima?
- How did you find a place to live?
- Do you think it’s possible you will move elsewhere in the future?

1. **Step 2 Interview**

## Review of the previous interview

The interviewer will show the participants their individual summaries of the previous narrative: “We’ve organized what you talked about last time, and it looks like this. Are there any points that you found strange or interesting, or that you would like to know more about?”

## Updated questions: The researchers updated the questions after an interim analysis of the previous interview narratives, and they added some questions raised during the analysis. For example:

- Did you predict that you would have these problems before you moved here?
- What feelings (wishes, expectations) do you think caused these difficulties?
- What do you feel would make your life in Fukushima more enjoyable and better?
- Which of these problems do you think are the most important (painful, unbearable)?
- If you were to leave Fukushima in the future, what reasons would you give?

## Additional questions: The researchers added questions about needs found in migration in general. For example:

- Do you value the fact that there are many other immigrants in the area you migrated to?
- Have you experienced any economic changes because of your migration?
- Have you been harassed by locals?

1. **Step 3 Interview**

## Review of the previous interview

The interviewer presents each participant with the results of the analysis of all the participants’ narratives and explains them in detail. “Now that you have seen it like this, is there anything strange or interesting, or something you would like to know more about?”

## Updated questions: The researchers updated the questions after an interim analysis of the previous interview narratives, and they added some questions raised during the analysis.

## Final questions about migration

- - - What did you gain or lose by moving to Fukushima compared with if you had stayed in your hometown?
    - What do you think you would have missed out on if you had not moved to Fukushima?
    - When exactly did you start thinking about settling down (in what year)?
    - What was the experience of migration like for you? What are your thoughts on moving to a place where there was a major disaster?

# End of Interview

At the end of the interviews, interviewers should express their appreciation for the participants’ cooperation with the study. They should ask if the participants have any questions about the interviews.
